# Biopersistence of PEGylated Carbon Nanotubes Promotes a Delayed Antioxidant Response after Infusion into the Rat Hippocampus

**DOI:** 10.1371/journal.pone.0129156

**Published:** 2015-06-15

**Authors:** Lidiane Dal Bosco, Gisele E. Weber, Gustavo M. Parfitt, Arthur P. Cordeiro, Sangram K. Sahoo, Cristiano Fantini, Marta C. Klosterhoff, Luis Alberto Romano, Clascídia A. Furtado, Adelina P. Santos, José M. Monserrat, Daniela M. Barros

**Affiliations:** 1 Programa de Pós-graduação em Ciências Fisiológicas–Fisiologia Animal Comparada, Instituto de Ciências Biológicas, Universidade Federal do Rio Grande, Rio Grande, RS, Brazil; 2 Escola de Química e Alimentos, Universidade Federal do Rio Grande, Rio Grande, RS, Brazil; 3 Instituto de Ciências Exatas, Departamento de Física, Universidade Federal de Minas Gerais, Belo Horizonte, MG, Brazil; 4 Programa de Pós-graduação em Aquicultura, Instituto de Oceanografia, Universidade Federal do Rio Grande, Rio Grande, RS, Brazil; 5 Laboratório de Química de Nanoestruturas, Centro de Desenvolvimento da Tecnologia Nuclear, Belo Horizonte, MG, Brazil; Indian Institute of Integrative Medicine, INDIA

## Abstract

Carbon nanotubes are promising nanomaterials for the diagnosis and treatment of brain disorders. However, the ability of these nanomaterials to cross cell membranes and interact with neural cells brings the need for the assessment of their potential adverse effects on the nervous system. This study aimed to investigate the biopersistence of single-walled carbon nanotubes functionalized with polyethylene glycol (SWCNT-PEG) directly infused into the rat hippocampus. Contextual fear conditioning, Y-maze and open field tasks were performed to evaluate the effects of SWCNT-PEG on memory and locomotor activity. The effects of SWCNT-PEG on oxidative stress and morphology of the hippocampus were assessed 1 and 7 days after infusion of the dispersions at 0.5, 1.0 and 2.1 mg/mL. Raman analysis of the hippocampal homogenates indicates the biopersistence of SWCNT-PEG in the hippocampus 7 days post-injection. The infusion of the dispersions had no effect on the acquisition or persistence of the contextual fear memory; likewise, the spatial recognition memory and locomotor activity were not affected by SWCNT-PEG. Histological examination revealed no remarkable morphological alterations after nanomaterial exposure. One day after the infusion, SWCNT-PEG dispersions at 0.5 and 1.0 mg/mL were able to decrease total antioxidant capacity without modifying the levels of reactive oxygen species or lipid hydroperoxides in the hippocampus. Moreover, SWCNT-PEG dispersions at all concentrations induced antioxidant defenses and reduced reactive oxygen species production in the hippocampus at 7 days post-injection. In this work, we found a time-dependent change in antioxidant defenses after the exposure to SWCNT-PEG. We hypothesized that the persistence of the nanomaterial in the tissue can induce an antioxidant response that might have provided resistance to an initial insult. Such antioxidant delayed response may constitute an adaptive response to the biopersistence of SWCNT-PEG in the hippocampus.

## Introduction

The ability of carbon nanotubes (CNT) to cross cell membranes and interact with neural cells make these nanomaterials promising for the development of drug delivery vehicles, gene delivery vectors and biomaterials for the diagnosis and treatment of brain disorders [1–5]. A fundamental step towards these applications is the evaluation of CNT neurotoxicity. Many studies have demonstrated the effects of CNT in primary neuro-glial cultures and PC12 neuronal cells [6–9]; however, there are few studies on the neurobehavioral changes that occur after nanomaterial exposure [10–12].

The pathogenic potential of CNT may be related to their ability to persist in biological systems despite clearance mechanisms, which is referred to as biodurability or biopersistence [13]. Although CNT are considered stable in biological environments, it has been reported that certain types of CNT are enzymatically biodegraded [14–16]. The degradation of amino-functionalized MWCNT also occurred after direct stereotactic injection into the motor cortex of mice [17], raising questions for further investigation on the consequences of nanomaterials biodegradation.

The aim of this study was to evaluate the biopersistence and neurotoxicity of SWCNT functionalized with PEG (SWCNT-PEG) 1 and 7 days after stereotaxic administration into the rat hippocampus. Raman spectroscopy was employed for the detection of SWCNT-PEG in the hippocampus and the effects of the nanomaterial on memory and locomotor activity were assessed by contextual fear conditioning, Y-maze and open-field tasks. Histological evaluation and oxidative stress analysis were carried out to evaluate potential biochemical and morphological changes in the hippocampus following SWCNT-PEG infusion.

## Material and Methods

### SWCNT-PEG dispersions

Single-walled carbon nanotubes functionalized with polyethylene glycol (SWCNT-PEG) were purchased from Sigma-Aldrich (St. Louis, MO, USA) and dispersed in deionized water employing mechanical disintegration and centrifugation steps, as recently described [12]. A complete physicochemical characterization of the same material has beenpreviously published [10, 12].

### Ethics statement

All experiments were performed in accordance with Brazil’s National Council for the Control of Animal Experimentation (CONCEA) guidelines and were authorized by the Ethics Committee for Animal Use of the Universidade Federal do Rio Grande (FURG; Permit Number: P029/2011).

### Animals

Adult male Wistar rats (2–3 months of age; weight 250–320 g) were obtained from the breeding colony at the Universidade Federal do Rio Grande (Rio Grande, RS, Brazil) and were randomly selected and housed in polycarbonate boxes containing a maximum of five animals per cage. The rats were kept under standard laboratory conditions (12 h light/dark cycle and a constant temperature of 23 ± 1°C) with free access to food and water and were frequently manipulated to avoid neophobia.

### Stereotaxic surgery and infusion of dispersions

Rats were allowed to adapt to the laboratory conditions 1 week before surgery. After this acclimation, the animals were anesthetized intraperitoneally with ketamine hydrochloride (50 mg/kg) and xylazine (4 mg/kg) and placed in a stereotaxic instrument for the bilateral implant of 22-gauge cannulae in the dorsal hippocampus using the following coordinates (in mm from Bregma):- 4.3 anteroposterior, ± 3.0 lateral,- 1.8 ventral, according to the atlas by Paxinos and Watson [18]. The guide cannulae were fixed with acrylic resin. At the end of the surgery the animals were treated with an antibiotic association (Pentabiótico, Fort Dodge, Brazil) to prevent infections.

After 48–72 h of recovery from surgery, the rats were distributed randomly into four experimental groups according to the treatments. The dispersions of SWCNT-PEG at 0.5, 1.0 or 2.1 mg/ml or 0.9% NaCl solution (control group) were infused using 27-gauge injection needles inserted into each guide cannula and connected by polyethylene tubing to a Hamilton microsyringe. The infusions, at a volume of 1 μL, were performed in one cannula at a time. All efforts were made to minimize animal suffering.

### Behavioral procedures

The study the effects of SWCNT-PEG dispersions on the acquisition and persistence of the fear memory, the animals were subjected to the contextual fear conditioning (CFC) task. The conditioning chamber (28 cm long, 26 cm high and 23 cm wide) was made of aluminum side walls and Plexiglas front wall. The floor consisted of a series of a parallel stainless steel bars connected to a shock scrambler delivery apparatus (shock generator, Insight Scientific Equipments, Brazil). The CFC procedure was carried out with training and test sessions, as previously described in [19]. During the training session, three consecutive electric foot-shocks (1 sec duration, 0.7 mA intensity) were applied, with 10-s intervals between each shock. The infusions of treatments were performed 30 min before training (acquisition group) and 12 h after training (persistence group). The test session was performed 1 day (acquisition group) or 7 days (persistence group) after training, and the freezing behavior (absence of any movement except that required for breathing) was quantified for 5 min. Both training and test sessions were performed between 8:00 and 12:00 a.m. The chambers were cleaned with 70% ethanol between each set of animals. The results are expressed as the percentage of time spent freezing in a 5 min test-session.

Another set of experiments was performed to assess the effects of SWCNT-PEG on locomotor activity and spatial recognition memory. The open field test was performed to evaluate locomotor activity. This task was performed in a square wood box (60 cm height x 40 cm width x 50 cm lenght) with the floor divided into 12 squares. The open field test was performed between 8:00 a.m. and 14:00 p.m. The rats were placed in the corner of the box and their behavior was monitored during 5 min. Six animals were assigned in each tested group. One group was tested 30 min and 1 day after the infusion and another just at 7 days post-injection. The apparatus was cleaned with 70% ethanol before each animal was tested. Total number of crossings (squares entered by the animals) was counted during a 5min period. All tests were recorded using a video camera to enable subsequent evaluation.

The Y-maze task was performed to evaluate spatial recognition memory. The single-session of Y-maze test was performed after the open field test at 1 and 7 days post-injection. The Y-maze apparatus used in this study was made of wood and consisted of three arms (40 cm long, 20 cm high and 12 cm wide) at a 120° angle from each other. The rats were placed in the center of the maze and were allowed to move freely for 10 min. The series of arm entries were recorded visually by an independent observer blind to treatments. An arm entry was counted when the hind paws of the rat are completely within the arm. Spontaneous alternation was defined as successive entries into the three arms on overlapping triplet sets, as described in [20]. The number of alternations opportunities was defined as the total number of arms entries minus 2. The percentage of alternation was calculated as the number of alternations divided by the number of alternation opportunities plus 100. The apparatus was cleaned with 70% ethanol before each animal was tested.

### Tissue dissection and sample preparation

All animals were killed by decapitation following the guidelines of CONCEA. For the analysis of oxidative stress and Raman spectroscopy, the hippocampi were quickly dissected at the end of the CFC test and stored at -80°C until use, except for those needed to measure ROS, which were immediately homogenized (1:5 w/v) in 40 mM ice-cold Tris-HCl buffer (pH 7.4). For the analysis of antioxidant capacity against peroxyl radicals (ACAP), glutamate cysteine ligase (GCL) activity and glutathione (GSH), hippocampi were kept on ice and homogenized in buffer containing 100 mM Tris-HCl, 2 mM EDTA and 5 mM MgCl_2_ (pH 7.75). Then, the tissue homogenates were centrifuged at 10,000 *g*, 4°C, for 20 min. For LPO analysis, the hippocampi were homogenized (1:15 w/v) in 100% ice-cold methanol and centrifuged at 1000 g for 10 min at 4°C. For Raman spectroscopy, the hippocampi were homogenized (1:4 w/v) in lyses buffer (1% SDS, 1% Triton X-100, 40 mM Tris acetate, 10 mM EDTA, 10 mM DTT) using a tissue homogenizer. Shortly before the Raman spectroscopy analysis, the tissue homogenates were heated at 70°C for two hours to obtain a clear lysate.

### Raman spectroscopy of hippocampal homogenates

The Raman spectroscopy analysis was performed in a Horiba T64000 Raman spectrometer (laser excitation wavelength = 785 nm). At least three spectra were taken for each sample for averaging. The tissue samples from animals exposed to nanomaterial and positive and negative controls were analyzed. The positive control was made by mixing 1 μL of SWCNT-PEG dispersion at 0.5 with 200 μL of lyses buffer. The negative control was made by using the hippocampal homogenate from a control animal, i.e., a rat that received an infusion of 1 μL of saline solution (0.9% NaCl). At least 50 μL of each sample was used for obtaining the Raman spectra. To determine the limit of detection of the SWCNT-PEG, a detection curve was generated by diluting the dispersion at 0.5 mg/mL.

### Histological analysis

For the histological examination, the brains were dissected immediately after the Y-maze test and fixed on Bouin solution for 12 h at room temperature. Tissues were thentransferred to 70% ethanol to be processed for routine histology. Brain specimens were embedded in paraffin, serially sectioned at 5 μm and stained with hematoxylin and eosin (H&E). Coronal sections were observed and examined under light microscope (Zeiss Primo Star) and the images were registered by a digital camera (Zeiss AxioCam ERc 5s).

### Measurement of oxidative stress parameters

Sample preparation was carried out as previously described. After homogenization in the buffer, the total protein content of the supernatants was measured via the Biuret method using a commercial kit (Doles, Goiânia, GO, Brazil) and a microplate absorbance reader (BioTek LX 800). The final protein concentration was adjusted to 3 mg/mL. The oxidative stress evaluation was performed by determining the concentration of reactive oxygen species (ROS), total antioxidant capacity against peroxyl radicals (ACAP), content of reduced glutathione (GSH), activity of glutamate-cysteine ligase (GCL) and levels of lipid peroxidation (LPO).

ROS concentration was quantified using the compound 2′,7′-dichlorofluorescein diacetate (H_2_DCF-DA, Molecular Probes Eugene, OR, USA), as previously employed for brain tissue [21, 22]. Briefly, the samples were placed in reaction buffer (pH 7.2) containing 200 mM KCl, 30 mM HEPES, 1 mM MgCl_2_ and 16 μM H_2_DCFDA. Using a fluorescence microplate reader (485 nm excitation/520 nm emission; Victor 2, Perkin Elmer), the formation of the oxidized fluorescent product dichlorofluorescein (DCF) was monitored with readings every 5 min for 30 min. ROS generation was calculated by integrating the fluorescent units along the time of measurement and after fitting the data to a second order polynomial function and was expressed in area.

ACAP determination employed the quantification of ROS using H_2_DCFDA (40 μM final concentration). Hippocampus samples were treated or not with 4 mM 2,2′-azobis(2-methylpropionamidine) dihydrochloride (ABAP, Sigma-Aldrich, St. Louis, MO, USA), a substrate that generates peroxyl radicals through thermal decomposition. DCF fluorescence was recorded by a fluorescence microplate reader (485 nm excitation/520 nm emission; Victor 2, Perkin Elmer) with readings every 5 min for 30 min. The inverse of relative difference between ROS area with and without ABAP was considered as a measure of antioxidant capacity. The protocol was performed following the methodology described in [23].

GSH content and GCL activity were determined by the method based in the reaction of naphthalene-2,3-dicarboxaldehyde (NDA, Molecular Probes Eugene, OR, USA) with GSH or γ-glutamyl cysteine (γ-GC) to form cyclic products that are highly fluorescent [24]. NDA-GSH fluorescence (485 nm excitation/530 nm emission) was measured using a fluorescence microplate reader (Victor 2, Perkin Elmer). GSH content is expressed in μM/mg of proteins, and GCL activity is expressed in μM/min/mg of proteins.

LPO was determined by a spectrophotometric assay of the ferrous oxidation/xylenol orange (FOX) modified method as previously described in [25], with adjustments in time of incubation and sample dilution according [21]. The basic reaction of this method is the oxidation of Fe(II) under acidic conditions and the quantification of lipid hydroperoxides using 0.1 mM cumene hydroperoxide (CHP, Sigma-Aldrich, St. Louis, MO, USA) as a standard. CHP absorbance (550 nm) was determined using a microplate reader (BioTek LX 800), and the results are expressed as nM of CHP per gram of wet tissue.

Fluorescence-based *in vitro* assays were carried out to verify the potential interference of SWCNT-PEG with DCF and NDA-GSH fluorescence. For these, 1 μL of distilled water or SWCNT-PEG dispersions at 0.5, 1.0 or 2.1 mg/mL were directly added to 300 μL of hippocampus extracts (protein concentration adjusted to 3 mg/mL) obtained from naïve animals. These samples were immediately subjected to ACAP and GSH measurements as described above. The results of the ACAP *in vitro* assay are expressed as ROS area with and without ABAP. The results of the *in vitro* GSH assay are expressed in μM/mg of proteins. The dilution of SWCNT-PEG used in these assays (1:300 v/v) were calculated from the higher estimated concentration of SWCNT-PEG that could remain on the rat hippocampus after processing, considering the average weight of 60 μg per hippocampus and the volume of buffer (1:5 w/v) used for tissue homogenization.

### Statistical analysis

One-way statistical analysis of variance (ANOVA) was employed to assess the statistical significance of the results. Post hoc analysis was carried out by Newman-Keuls multiple comparisons tests, when appropriate. P-values <0.05 were considered statistically significant. The results of the oxidative stress assays were normalized to the percentage of the control group. All data were expressed as mean ± SEM.

## Results and Discussion

### Behavioral parameters

The hippocampus is a complex structure of the limbic system that plays an important role in spatial navigation and episodic memory [26]. Behavioral tasks involving associative learning and remembering contexts have been widely used for the study of hippocampal function in animals [27, 28]. In this work, the effect of SWCNT-PEG infused into the rat hippocampus on memory function was assessed by two paradigms: spontaneous alternation in the Y maze and time spent in freezing in the contextual fear conditioning task. The open field test was used to evaluate possible interference of the treatments on locomotor activity that might have affected the performance of the rats in memory tests.

**Fig 1:**
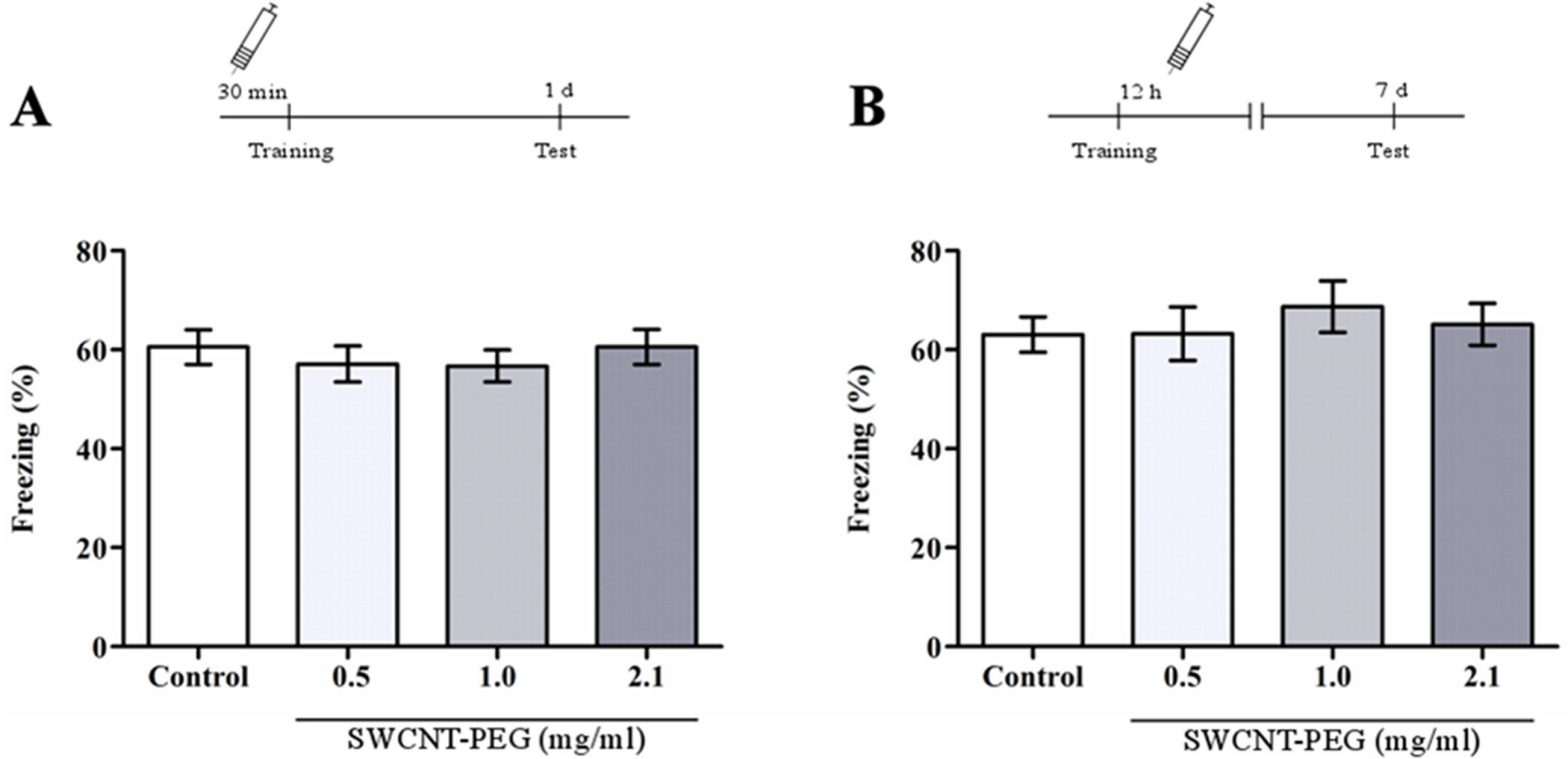
Effect of SWCNT-PEG dispersions on (A) acquisition and (B) persistence of contextual fear memory. Schematics of the procedures used in the experiments are presented above the graphs. Values are expressed as the mean ± SEM, n = 10–12. No significant difference in time spent in freezing was observed between the groups (*p*>0.05).

The infusion of the treatments made before the training session in the CFC task allowed us to evaluate if the nanomaterial affects the acquisition, i.e., the first stage of memory processing in which an association is established between the context and the shock [29]. The infusion performed 12 h post-training aimed to evaluate if the SWCNT-PEG could impair cellular and molecular late events that occur in the rat hippocampus at this time point and are required for fear memory persistence 7 days after conditioning [19]. Our results showed that the SWCNT-PEG dispersions had no effects on the acquisition and persistence of the contextual fear memory (Fig 1), as indicated by similar time spent freezing between the treated animals and the control group.

We also evaluated the effect of SWCNT-PEG dispersions on spontaneous alternation behavior in the Y-maze. Spontaneous alternation constitutes an unlearned response that provides information about exploratory behavior, perception, attention and spatial memory of rodents [20]. Y-maze task has been widely used to study the effects of different experimental conditions on cognitive functions [20, 30, 31].

**Fig 2:**
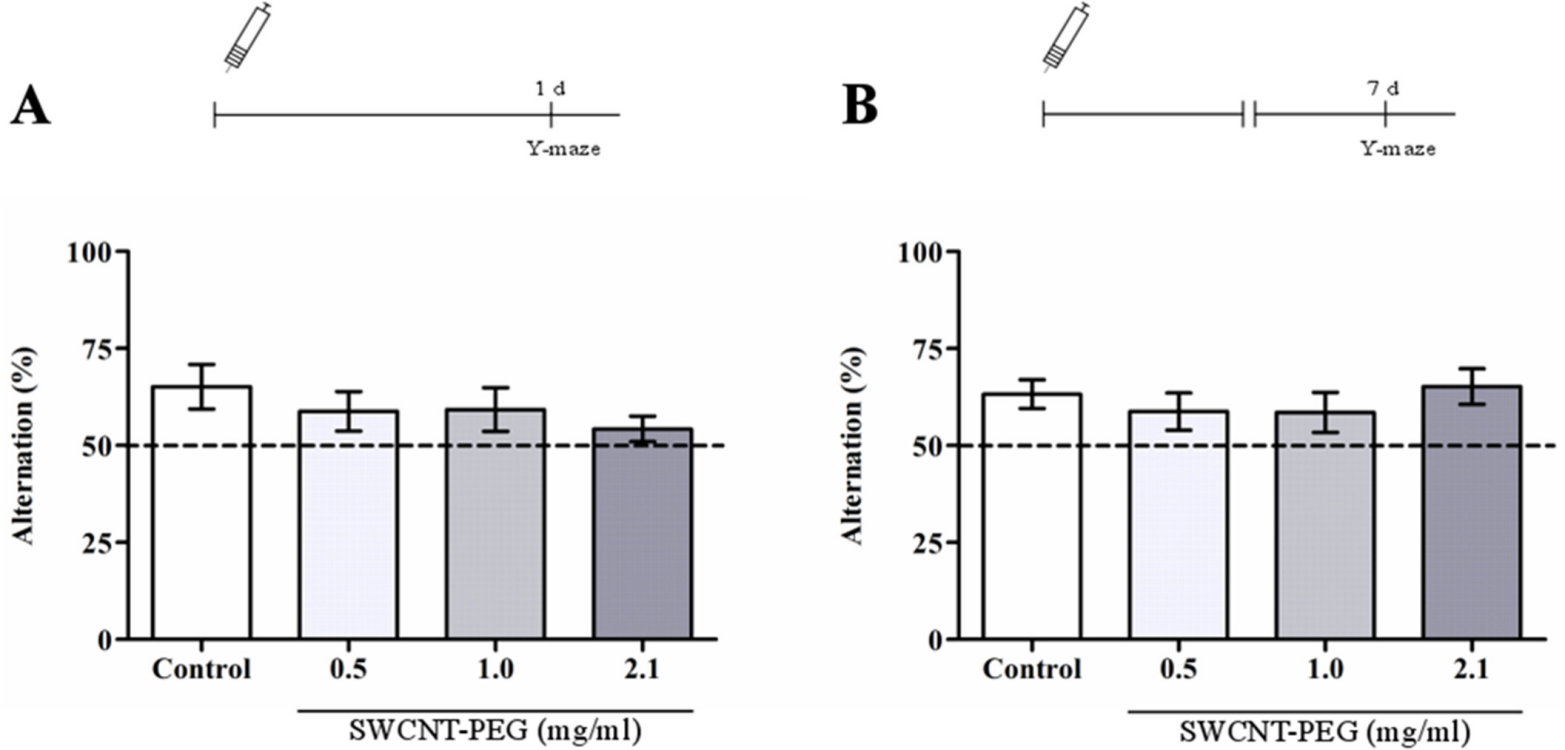
Effect of SWCNT-PEG dispersions on spontaneous alternation behavior (A) 1 and (B) 7 days after infusion. Schematics of the procedures used in the experiments are presented above the graphs. Values are expressed as the mean ± SEM, n = 6. No significant difference in percentage of alternation was observed between the groups (*p*>0.05).

In this work rats were tested in Y-maze task after the open-field test was conducted. It was demonstrated that SWCNT-PEG did not affect the spontaneous alternation behavior 1 and 7 days after the infusion of dispersions (Fig 2). The effect of the treatments on the locomotor activity was evaluated 30 min, 1 and 7 days after infusion, and no differences between SWCNT-PEG and control animals were observed at these time-points (Table 1). These results were in agreement with the total number of entries in the Y-maze arms (*data not shown*), confirming that SWCNT-PEG exposure had no effect on locomotor activity of rats.

**Table 1:** Effect of SWCNT-PEG on the number of crossings in open field task.

Treatment	Time after infusion		
	30 min	1 day	7 days
Saline (control group)	85.50 ± 12.92	56.17 ± 12.14	77.33 ± 8.29
SWCNT-PEG 0.5 mg/mL	65.80 ± 13.47	65.50 ± 9.88	65.67 ± 9.85
SWCNT-PEG 1.0 mg/mL	68.00 ± 9.99	43.75 ± 8.79	84.50 ± 7.41
SWCNT-PEG 2.1 mg/mL	82.00 ± 10.72	65.83 ± 6.84	87.50 ± 11.49

Values expressed as the mean ± SEM (n = 6). Animals tested at 30 min were subjected to another test-session at 1 day. No significant differences were observed between the groups (*p*>0.05).

### Raman spectroscopy analysis

Raman spectroscopy is a molecular vibrational spectroscopy that provides important information about CNT structure and purity [32, 33]. The specificity of this technique allows the accurate detection of CNT in complex biological systems and has been widely used to probe the biodistribution of SWCNT in organs and tissues from different animal models [10, 34, 35]. In this study, we employed a Raman spectroscopy analysis to verify how the CNT are functionalized and to detect SWCNT-PEG in the hippocampus 1 and 7 days after stereotactic administration.

**Fig 3:**
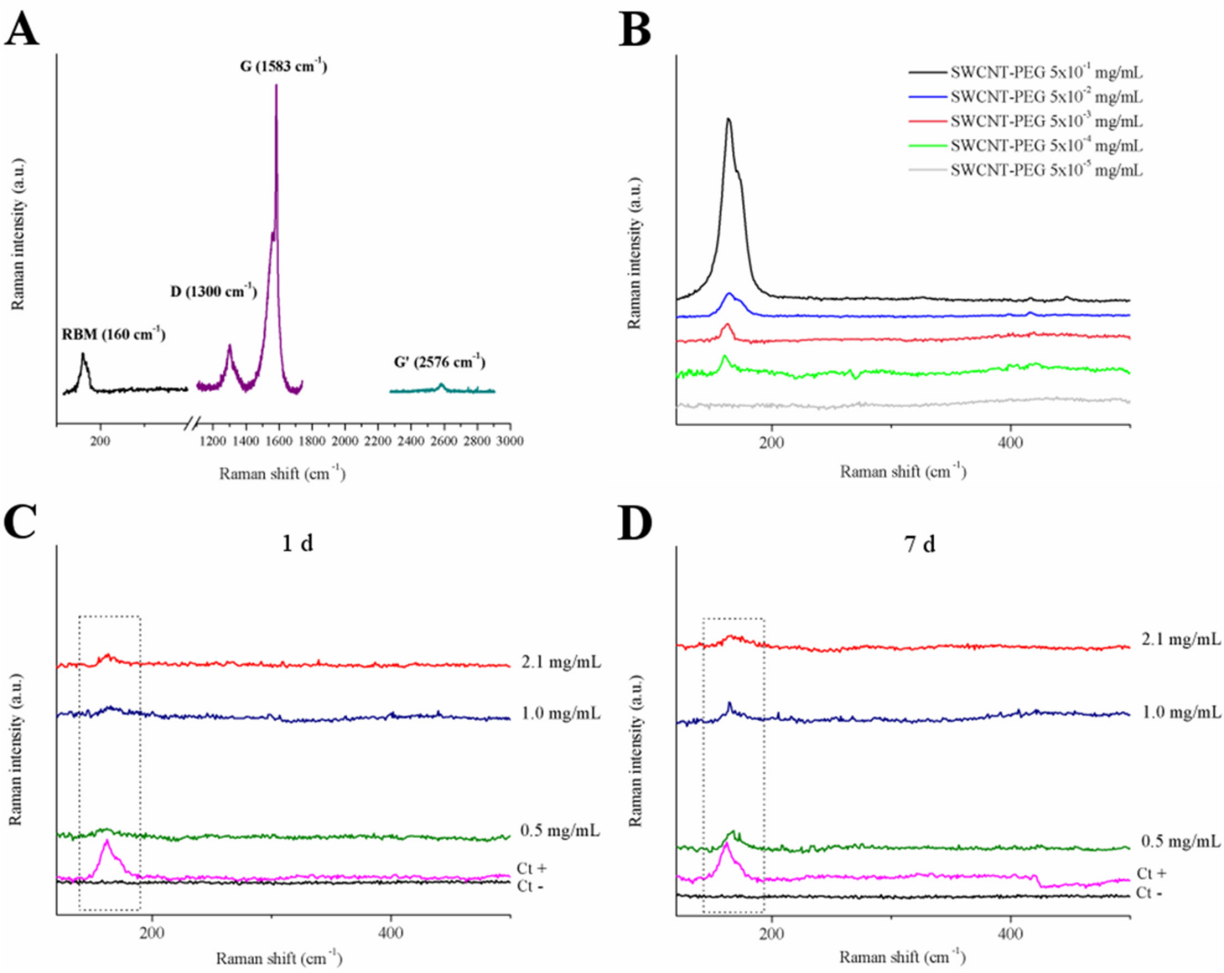
Raman spectroscopy of rat hippocampal homogenates 1 and 7 days after SWCNT-PEG dispersions infusion. (A) Raman spectrum acquired from SWCNT-PEG dispersion at 2.1 mg/mL. (B) Detection curve of SWCNT-PEG based on radial breath mode (RBM). Raman spectra in hippocampal homogenates (C) 1 and (D) 7 days after infusion. Dotted squares indicate the RBM region. Ct+: positive control; Ct-: negative control.

In the analysis of the Raman spectrum of SWCNT-PEG, the vibrational modes observed were the radial breathing mode (RBM), D band and G band (Fig 3A). The presence of a well-defined and intense G band confirms that the functionalization of SWCNT occured in the bundles rather than in individual CNT, as previously reported for the same material [10, 12]. The detection of SWCNT-PEG in the hippocampal homogenates was determined based on the RBM, a Raman mode unique for SWCNT. The position and shape of the RBM, generally between 140 and 220 cm^-1^, depends strongly on the exciting laser wavelength and can be affected by the intertube interaction in the bundles [36].

The detection limit of this assay was found to be 5x10^-5^ mg/mL (Fig 3B). The detection of the RBM peak in the tissue homogenates indicates the presence of SWCNT-PEG both 1 and 7 days after their infusion into the rat hippocampus (Fig 3C and 3D). This result showed that at least part of the SWCNT-PEG infused into the hippocampus remained after 7 days. However, it cannot rule out the possibility that a certain amount of material has been modified or degraded. Besides that, it should consider that more time might be required for the complete biodegradation of SWCNT-PEG in the hippocampus.

It was reported that the oxidative biodegradation of SWCNT in lungs of mice increased over time and can take several days [37], whereas the degradation of amino functionalized MWCNT in mice brain can start within 2 days post-injection [17]. Such differences may reflect the interaction of many factors in the biodegradation process, such as the enzymatic profile of the tissue and the type of surface functionalization of the nanomaterial. Lastly, it is important to consider that if on the one hand the biodegradation of CNT can facilitate the elimination and reduce toxicity [15], on the other hand it can generate degradation products, such as oxidized aromatic hydrocarbons [14], that could cause unpredicted toxicity. Thus, the consequences of the biopersistence of CNT in the organs and tissues of living organisms should be evaluated carefully.

### Histological assessment

**Fig 4:**
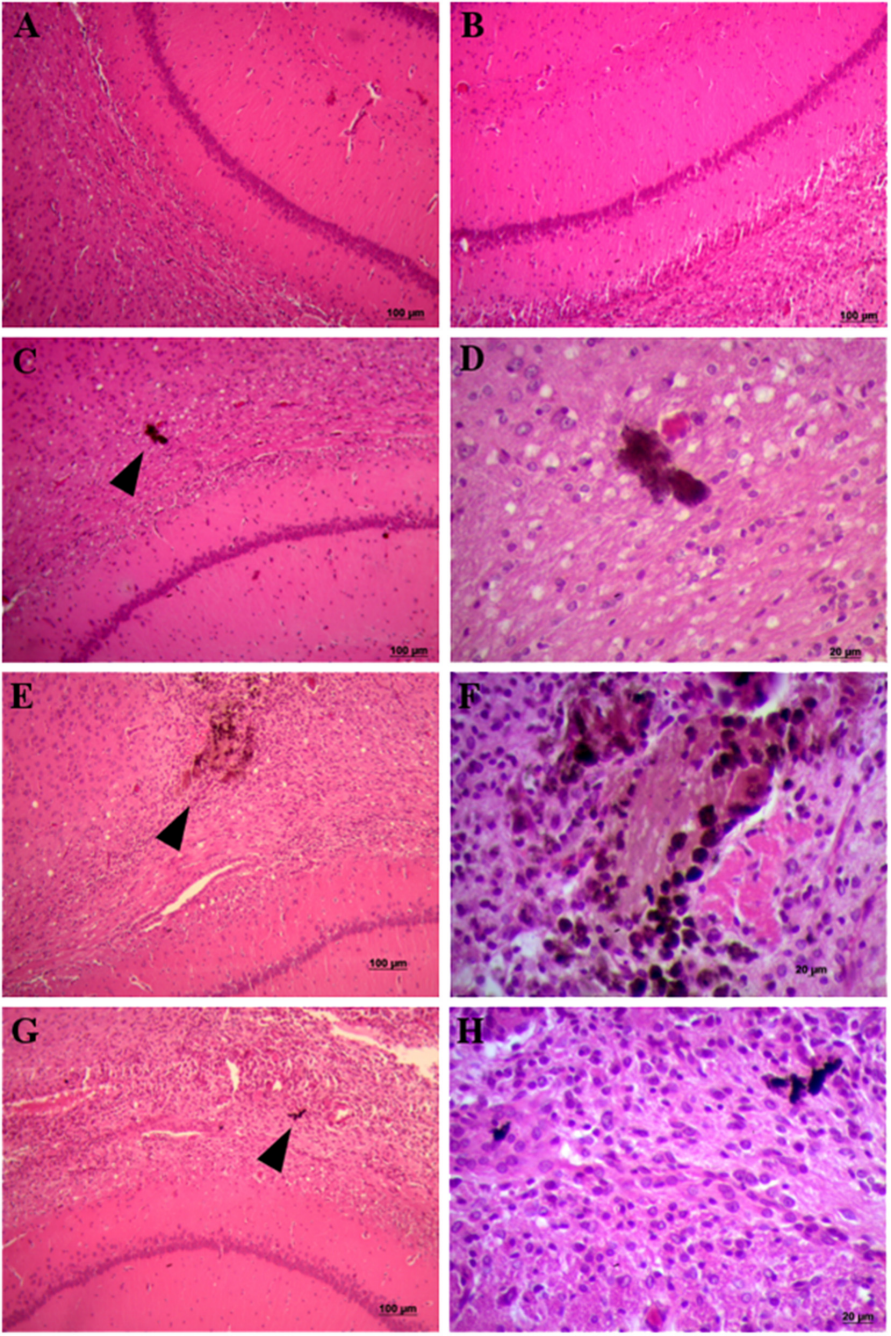
Histological analysis of hippocampus of rats infused with saline solution and SWCNT-PEG dispersions. Representative images of brain coronal sections of control animals (A) 1 and (B) 7 days after the infusion of saline solution. Representative images of brain coronal sections of animals treated with SWCNT-PEG dispersions (C, D) 1 and (E-H) 7 days after infusion. (C, E, G) Black arrowheads indicate the presence of SWCNT-PEG in the brain parenchyma. (D, F, H) High magnification of SWCNT-PEG in the injected tissue.

Histological examination of brain coronal sections was performed 1 and 7 days after the stereotactic administration of SWCNT-PEG dispersions to evaluate potential morphological changes due to the presence of the nanomaterial in the nervous tissue. Brain sections of control animals were also examined (Fig 4A and 4B). It was observed a mild inflammation in the regions where guide cannulae were implanted. The infiltration of inflammatory cells was consistent with a localized tissue reaction at the site of cannulae implantation. SWCNT-PEG agglomerates were observed in the cortical area adjacent to dorsal hippocampus in treated animals (Fig 4C–4H). No remarkable morphological alterations such as cells with neuronal distress, cells with satellitosis, glial proliferation and brain edema were observed. By the histological assessment we can infer that direct SWCNT-PEG infusion did not induce cell death in the hippocampus and adjacent areas.

### Oxidative stress parameters

Considering that it was previous observed that SWCNT-PEG exposure can modify oxidative stress parameters even in the absence of cognitive deficits [12] and to further explore the potential adaptative responses to the presence of SWCNT-PEG in the hippocampus, we investigated ROS, LPO and antioxidant levels. The brain contains multiple antioxidant defenses, among them GSH is especially important because of its activity as ROS scavenger and contribution to the cellular redox state maintenance [38]. Besides that, the determination of the integrated antioxidant response through the measurement of total antioxidant capacity may be useful because it provides a general scenario of the oxidative status of the tissue [23].

**Fig 5:**
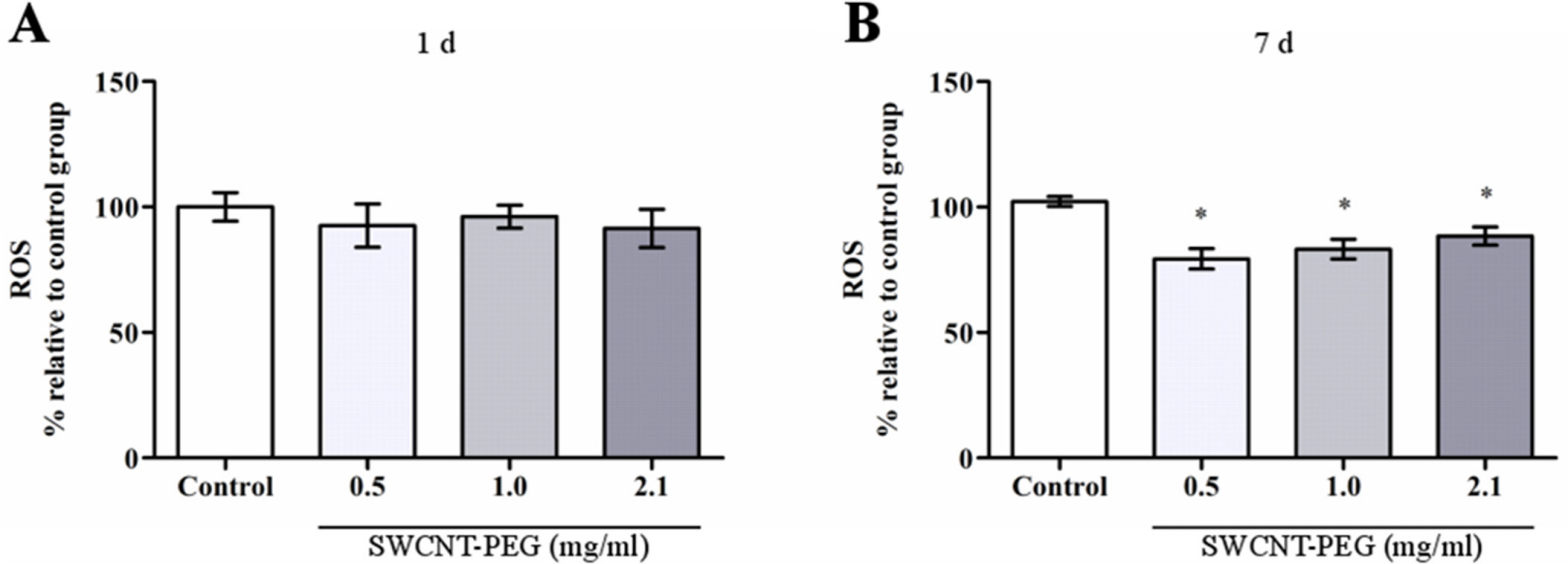
Levels of reactive oxygen species (ROS) in the hippocampus (A) 1 and (B) 7 days after theinfusion of SWCNT-PEG dispersions. Values are expressed as the mean ± SEM, n = 4–6. *p <0.05 vs. control group.

**Fig 6:**
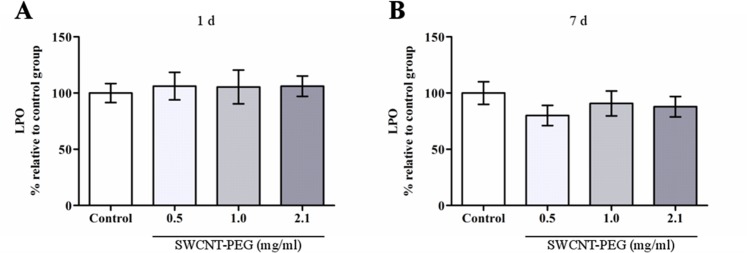
Effect of SWCNT-PEG dispersions on lipid peroxidation (LPO) in the hippocampus (A) 1 and (B) 7 days after infusion. Values are expressed as the mean ± SEM, n = 4–6. No significant difference in LPO levels was observed between the groups (*p*>0.05).

**Fig 7:**
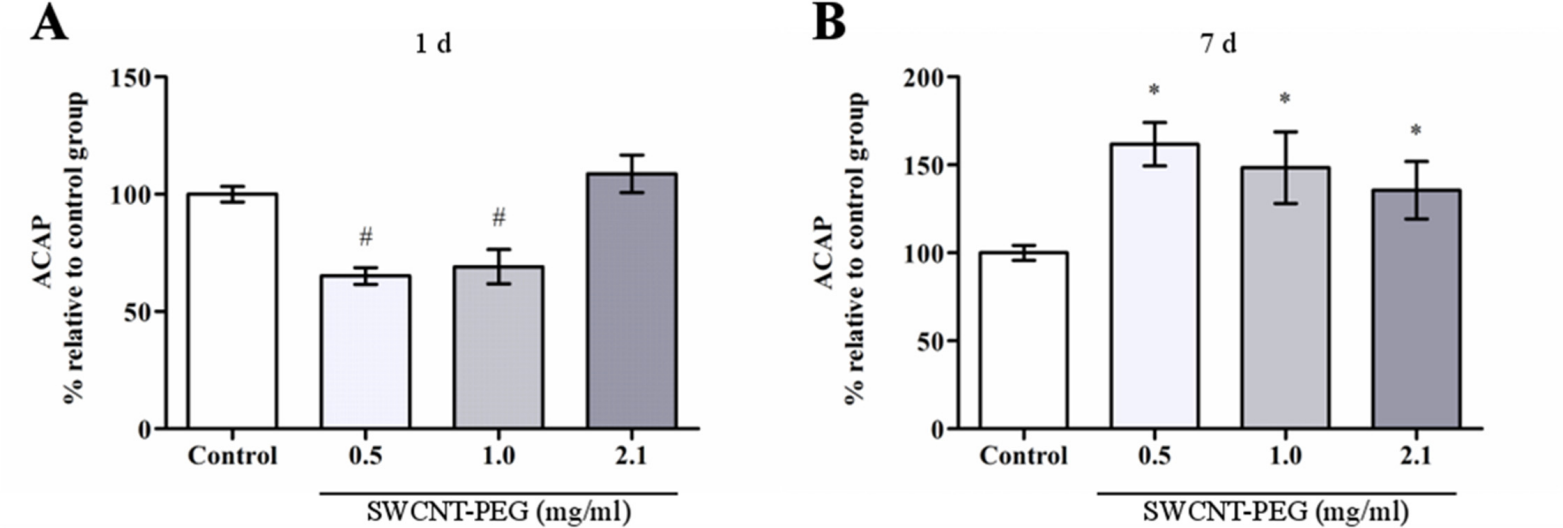
Effect of SWCNT-PEG dispersions on total antioxidant capacity against peroxyl radicals (ACAP) in the hippocampus (A) 1 and (B) 7 days after infusion. Values are expressed as the mean ± SEM, n = 4–6. ^#^ *p*<0.05 vs. 2.1 mg/mL SWCNT-PEG and vs. the control group; *p <0.05 vs. the control group.

**Fig 8:**
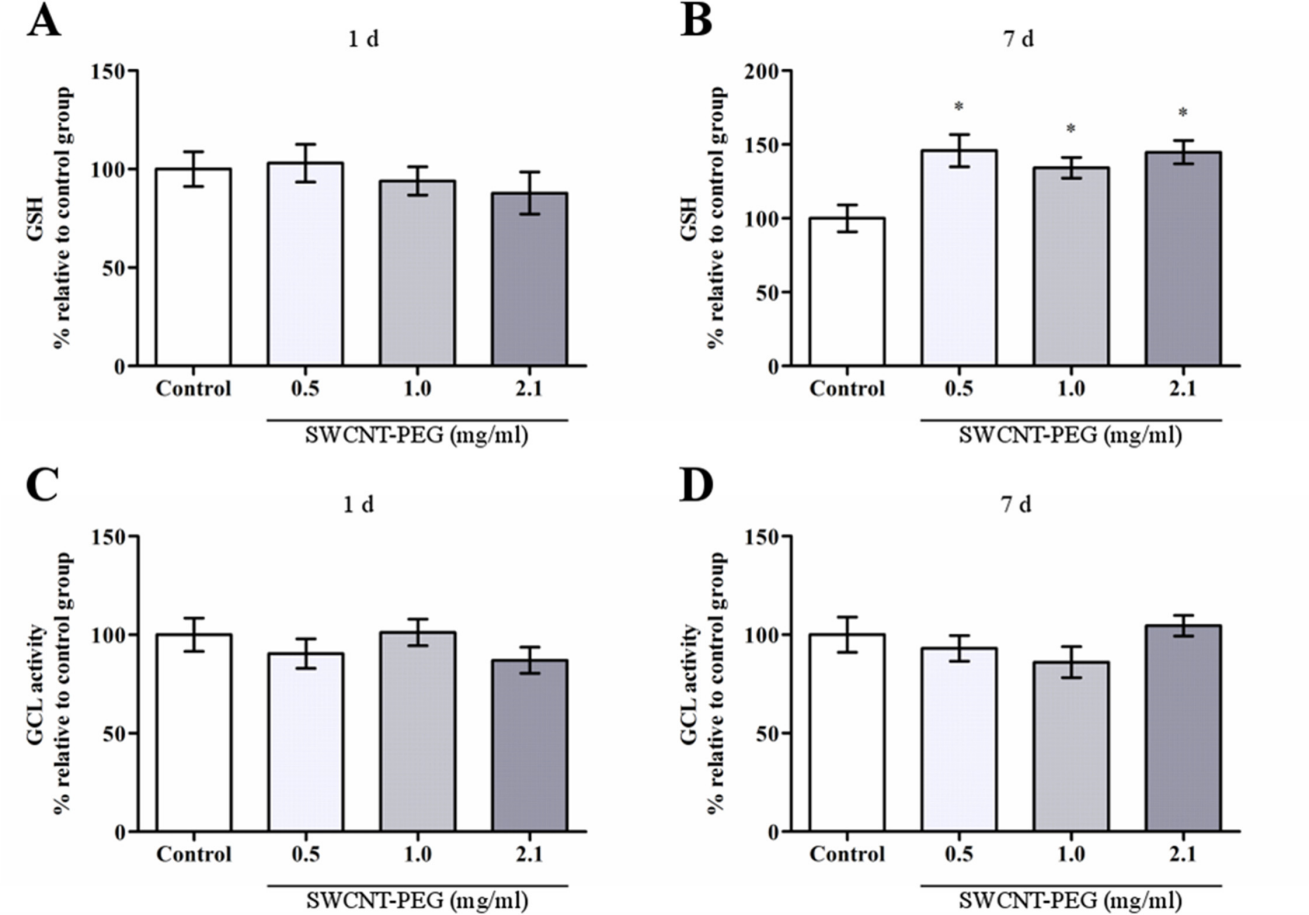
Glutatione (GSH) content and glutamate cysteine-ligase (GCL) activity in the hippocampus after the infusion of SWCNT-PEG dispersions. (A, B) GSH content 1 and 7 days after the infusion. (C, D) GCL activity 1 and 7 days post-injection. Values are expressed as the mean ± SEM, n = 4–6. *p <0.05 vs. the control group.

In this work, we found that ROS concentration in the hippocampus was not altered 1 day after the infusion of SWCNT-PEG dispersions (Fig 5A), on the other hand, ROS levels were decreased 7 days after the infusion (Fig 5B). Lipid hydroperoxide levels were unaltered at both time-points (Fig 6). Regarding antioxidant defenses, ACAP was lowered 1 day after the infusion of dispersions at 0.5 and 1.0 mg/mL (Fig 7A), but was increased 7 days after the infusion of the dispersions at 0.5, 1.0 and 2.1 mg/mL (Fig 7B). Similarly, there was an increase in GSH content 7 days after the infusion of SWCNT-PEG dispersions (Fig 8B), which may have contributed to the higher ACAP values registered. However, any changes in GCL activity occurred at this time point (Fig 8D).

The biopersistence of SWCNT-PEG over time may have an important effect on the antioxidant response that culminates in increased ACAP and GSH content in the hippocampus, resulting in lower ROS levels. A decrease in ROS production was previously observed in the lungs after double-walled CNT instillation [39]. Such an effect was attributed to the ROS scavenger capability of CNT and based on the assumption from that certain ROS types may be readily linked at the surface of CNT by mechanisms similar to the grafting of organic functionalities [40]. However, the decrease in ROS production found in this work may not be attributed to the scavenging action of SWCNT-PEG because the *in vitro* ROS and ACAP assays did not show any intrinsic antioxidant activity, as discussed below.

Based on the evidence that the fluorescence of DCF can be partially quenched by SWCNT [41], we performed fluorescence-based *in vitro* assays to ensure that the changes in ROS, ACAP and GSH found 7 days after the infusion of SWCNT-PEG resulted from the biological response to nanomaterial exposure. The results from the fluorescence-based *in vitro* assays are summarized in Table 2. The SWCNT-PEG dispersions did not quench DCF-induced fluorescence, both in the presence and in the absence of the peroxyl radicals generator ABAP. Besides that, no interference of SWCNT-PEG was observed in NDA-GSH fluorescence generation. Thus, we can discard any interference of the SWCNT-PEG in the determination of GSH content. These results allow the interpretation of the aforementioned measurements from *in vivo* biochemical assays as reliable biological responses and not related to SWCNT-PEG interference in the fluorescence-based assays.

**Table 2:** Fluorescence-based *in vitro* assays.

SWCNT-PEG dispersion	ROS area without ABAP	ROS area with ABAP	GSH (μM/mg protein)
0 mg/mL	207000 ± 13350	1176000 ± 18240	224.0 ± 22.01
0.5 mg/mL	227900 ± 16530	1139000 ± 38350	236.1 ± 25.05
1.0 mg/mL	218600 ± 17318	1238000 ± 73450	233.2 ± 28.96
2.1 mg/mL	229100 ± 20250	1134000 ± 78140	222.9 ± 24.94

Values are expressed as the mean ± SEM (n = 4). No significant differences were registered for the different SWCNT-PEG concentrations (*p*>0.05).

Here, we found a time-dependent change in antioxidant defenses, suggesting that SWCNT-PEG exposure can induce an antioxidant response in the hippocampus that may confer resistance or adaptation to the initial insult. Furthermore, these higher antioxidant levels may have contributed to the biopersistence of SWCNT-PEG in the hippocampus, since the addition of the antioxidants ascorbic acid and GSH suppressed the *in vitro* biodegradation of oxidized-SWCNT induced by hypochlorite and myeloperoxidase [42].

The higher GSH contents 7 days post-injection may constitute an important mechanism to maintain functional and structural integrity of the hippocampus after SWCNT-PEG exposure. A variety of different compounds increase GSH levels in cells by increasing the activity of glutamate cysteine ligase (GCL), the enzyme that catalyzes the first and rate-limiting step in the synthesis of GSH [43]. The transcription factor nuclear factor erythroid 2-related factor 2 (Nrf2) controls the basal and inducible expression of genes encoding the catalytic and regulatory chains of GCL. In response to oxidative stress, Nrf2 translocates to the nucleus and binds to antioxidant response elements, inducing the transcription of GCL and other cytoprotective enzymes [44].

The indirect antioxidant activity of a carbon nanomaterial was demonstrated by the pretreatment of cells with a polyhydroxylated fullerene derivative that was able to restore the Nrf2 expression level after a neurotoxic insult [45]. The increased expression of Nrf2 was also reported in the brains of zebrafish after systemic exposure to SWCNT [46]. These studies demonstrated the ability of carbon nanomaterials to induce antioxidant defenses and to prevent a potential damage to the neural tissue.

## Conclusion

This study showed that SWCNT-PEG were able to persist in the rat hippocampus 7 days after infusion. The exposure to this nanomaterial did not induce cognitive impairments or hippocampal damage after 1 and 7 days. Interestingly, the exposure to SWCNT-PEG was able to induce antioxidant defenses, possibly after an initial pro-oxidant effect. The delayed antioxidant response observed in this study may constitute an adaptive response to SWCNT-PEG biopersistence, which was associated with high GSH content that may provided protection against a putative initial oxidative damage and prevented the biodegradation of the nanomaterial in the tissue. Histological examination was in agreement with biochemical and behavioral findings. However, further studies on the gene expression and cell signaling pathways are needed to elucidate the mechanisms that may confer protection to the nervous tissue after SWCNT-PEG exposure.

## References

[1] Cellot G, Ballerini L, Prato M, Bianco A. Neurons are able to internalize soluble carbon nanotubes: new opportunities or old risks? Small. 2010;6(23):2630–3. Epub 2010/09/23. 10.1002/smll.201000906 . 20859949

[2] Ren J, Shen S, Wang D, Xi Z, Guo L, Pang Z, et al The targeted delivery of anticancer drugs to brain glioma by PEGylated oxidized multi-walled carbon nanotubes modified with angiopep-2. Biomaterials. 2012;33(11):3324–33. Epub 2012/01/28. 10.1016/j.biomaterials.2012.01.025 . 22281423

[3] Al-Jamal KT, Gherardini L, Bardi G, Nunes A, Guo C, Bussy C, et al Functional motor recovery from brain ischemic insult by carbon nanotube-mediated siRNA silencing. Proc Natl Acad Sci U S A. 2011;108(27):10952–7. Epub 2011/06/22. 10.1073/pnas.1100930108 21690348 PMC3131324

[4] Lee HJ, Park J, Yoon OJ, Kim HW, Lee do Y, Kim do H, et al Amine-modified single-walled carbon nanotubes protect neurons from injury in a rat stroke model. Nat Nanotechnol. 2011;6(2):121–5. Epub 2011/02/01. 10.1038/nnano.2010.281 21278749 PMC4113082

[5] Roman JA, Niedzielko TL, Haddon RC, Parpura V, Floyd CL. Single-walled carbon nanotubes chemically functionalized with polyethylene glycol promote tissue repair in a rat model of spinal cord injury. J Neurotrauma. 2011;28(11):2349–62. Epub 2011/02/10. 10.1089/neu.2010.1409 21303267 PMC3218389

[6] Zhang Y, Xu Y, Li Z, Chen T, Lantz SM, Howard PC, et al Mechanistic toxicity evaluation of uncoated and PEGylated single-walled carbon nanotubes in neuronal PC12 cells. ACS Nano. 2011;5(9):7020–33. Epub 2011/08/27. 10.1021/nn2016259 . 21866971

[7] Wang J, Sun P, Bao Y, Dou B, Song D, Li Y. Vitamin E renders protection to PC12 cells against oxidative damage and apoptosis induced by single-walled carbon nanotubes. Toxicol In Vitro. 2012;26(1):32–41. Epub 2011/10/25. 10.1016/j.tiv.2011.10.004 . 22020378

[8] Meng L, Jiang A, Chen R, Li CZ, Wang L, Qu Y, et al Inhibitory effects of multiwall carbon nanotubes with high iron impurity on viability and neuronal differentiation in cultured PC12 cells. Toxicology. 2013;313(1):49–58. Epub 2012/12/12. 10.1016/j.tox.2012.11.011 . 23219591

[9] Fabbro A, Sucapane A, Toma FM, Calura E, Rizzetto L, Carrieri C, et al Adhesion to carbon nanotube conductive scaffolds forces action-potential appearance in immature rat spinal neurons. PLoS One. 2013;8(8):e73621 Epub 2013/08/21. 10.1371/journal.pone.0073621 23951361 PMC3741175

[10] Weber GE, Dal Bosco L, Goncalves CO, Santos AP, Fantini C, Furtado CA, et al Biodistribution and toxicological study of PEGylated single-wall carbon nanotubes in the zebrafish (*Danio rerio*) nervous system. Toxicol Appl Pharmacol. 2014;280(3):484–92. Epub 2014/08/30. 10.1016/j.taap.2014.08.018 . 25168427

[11] Liu X, Zhang Y, Li J, Wang D, Wu Y, Li Y, et al Cognitive deficits and decreased locomotor activity induced by single-walled carbon nanotubes and neuroprotective effects of ascorbic acid. Int J Nanomedicine. 2014;9:823–39. Epub 2014/03/07. 10.2147/ijn.s56339 24596461 PMC3930484

[12] Dal Bosco L, Weber GE, Parfitt GM, Paese K, Goncalves CO, Serodre TM, et al PEGylated Carbon Nanotubes Impair Retrieval of Contextual Fear Memory and Alter Oxidative Stress Parameters in the Rat Hippocampus. Biomed Res Int. 2015;2015:104135 Epub 2015/03/05. 10.1155/2015/104135 25738149 PMC4337111

[13] Liu X, Hurt RH, Kane AB. Biodurability of Single-Walled Carbon Nanotubes Depends on Surface Functionalization. Carbon N Y. 2010;48(7):1961–9. Epub 2010/03/31. 10.1016/j.carbon.2010.02.002 20352066 PMC2844903

[14] Allen BL, Kotchey GP, Chen Y, Yanamala NV, Klein-Seetharaman J, Kagan VE, et al Mechanistic investigations of horseradish peroxidase-catalyzed degradation of single-walled carbon nanotubes. J Am Chem Soc. 2009;131(47):17194–205. Epub 2009/11/07. 10.1021/ja9083623 . 19891488

[15] Kagan VE, Konduru NV, Feng W, Allen BL, Conroy J, Volkov Y, et al Carbon nanotubes degraded by neutrophil myeloperoxidase induce less pulmonary inflammation. Nat Nanotechnol. 2010;5(5):354–9. Epub 2010/04/07. 10.1038/nnano.2010.44 . 20364135 PMC6714564

[16] Vlasova II, Vakhrusheva TV, Sokolov AV, Kostevich VA, Gusev AA, Gusev SA, et al PEGylated single-walled carbon nanotubes activate neutrophils to increase production of hypochlorous acid, the oxidant capable of degrading nanotubes. Toxicol Appl Pharmacol. 2012;264(1):131–42. Epub 2012/08/14. 10.1016/j.taap.2012.07.027 . 22884993

[17] Nunes A, Bussy C, Gherardini L, Meneghetti M, Herrero MA, Bianco A, et al *In vivo* degradation of functionalized carbon nanotubes after stereotactic administration in the brain cortex. Nanomedicine (Lond). 2012;7(10):1485–94. Epub 2012/06/21. 10.2217/nnm.12.33 . 22712575

[18] Paxinos G, Watson C. The Rat Brain in Stereotaxic Coordinates 6th ed. Amsterdam: Elsevier Academic Press; 2007. 456 p.

[19] Bekinschtein P, Cammarota M, Igaz LM, Bevilaqua LR, Izquierdo I, Medina JH. Persistence of long-term memory storage requires a late protein synthesis- and BDNF- dependent phase in the hippocampus. Neuron. 2007;53(2):261–77. Epub 2007/01/17. 10.1016/j.neuron.2006.11.025 . 17224407

[20] Pioli EY, Meissner W, Sohr R, Gross CE, Bezard E, Bioulac BH. Differential behavioral effects of partial bilateral lesions of ventral tegmental area or substantia nigra pars compacta in rats. Neuroscience. 2008;153(4):1213–24. Epub 2008/05/06. 10.1016/j.neuroscience.2008.01.084 . 18455318

[21] de Aguiar RB, Dickel OE, Cunha RW, Monserrat JM, Barros DM, Martinez PE. Estradiol valerate and tibolone: effects upon brain oxidative stress and blood biochemistry during aging in female rats. Biogerontology. 2008;9(5):285–98. Epub 2008/04/04. 10.1007/s10522-008-9137-7 . 18386154

[22] Galhardi F, Mesquita K, Monserrat JM, Barros DM. Effect of silymarin on biochemical parameters of oxidative stress in aged and young rat brain. Food Chem Toxicol. 2009;47(10):2655–60. Epub 2009/08/04. 10.1016/j.fct.2009.07.030 . 19647779

[23] Amado LL, Garcia ML, Ramos PB, Freitas RF, Zafalon B, Ferreira JL, et al A method to measure total antioxidant capacity against peroxyl radicals in aquatic organisms: application to evaluate microcystins toxicity. Sci Total Environ. 2009;407(6):2115–23. Epub 2008/12/20. 10.1016/j.scitotenv.2008.11.038 . 19095287

[24] White CC, Viernes H, Krejsa CM, Botta D, Kavanagh TJ. Fluorescence-based microtiter plate assay for glutamate-cysteine ligase activity. Anal Biochem. 2003;318(2):175–80. Epub 2003/06/20. 10.1016/S0003-2697(03)00143-X . 12814619

[25] Monserrat JM, Geracitano LA, Pinho GL, Vinagre TM, Faleiros M, Alciati JC, et al Determination of lipid peroxides in invertebrates tissues using the Fe(III) xylenol orange complex formation. Arch Environ Contam Toxicol. 2003;45(2):177–83. Epub 2003/10/21. doi: 0.1007/s00244-003-0073. . 14565574 10.1007/s00244-003-0073-x

[26] Maren S, Phan KL, Liberzon I. The contextual brain: implications for fear conditioning, extinction and psychopathology. Nat Rev Neurosci. 2013;14(6):417–28. Epub 2013/05/03. 10.1038/nrn3492 . 23635870 PMC5072129

[27] Fanselow MS. Contextual fear, gestalt memories, and the hippocampus. Behav Brain Res. 2000;110(1–2):73–81. Epub 2000/05/10. . 10802305 10.1016/s0166-4328(99)00186-2

[28] Acheson DT, Gresack JE, Risbrough VB. Hippocampal dysfunction effects on context memory: possible etiology for posttraumatic stress disorder. Neuropharmacology. 2012;62(2):674–85. Epub 2011/05/21. 10.1016/j.neuropharm.2011.04.029 21596050 PMC3175276

[29] Abel T, Lattal KM. Molecular mechanisms of memory acquisition, consolidation and retrieval. Curr Opin Neurobiol. 2001;11(2):180–7. Epub 2001/04/13. . 11301237 10.1016/s0959-4388(00)00194-x

[30] Bagheri M, Joghataei MT, Mohseni S, Roghani M. Genistein ameliorates learning and memory deficits in amyloid beta(1–40) rat model of Alzheimer's disease. Neurobiol Learn Mem. 2011;95(3):270–6. Epub 2010/12/15. 10.1016/j.nlm.2010.12.001 . 21144907

[31] Pickering C, Alsio J, Morud J, Ericson M, Robbins TW, Soderpalm B. Ethanol impairment of spontaneous alternation behaviour and associated changes in medial prefrontal glutamatergic gene expression precede putative markers of dependence. Pharmacol Biochem Behav. 2015;132:63–70. Epub 2015/03/10. 10.1016/j.pbb.2015.02.021 . 25743187

[32] Dresselhaus MS, Dresselhaus G, Jorio A, Souza Filho AG, Samsonidze GG, Saito R. Science and applications of single-nanotube Raman spectroscopy. J Nanosci Nanotechnol. 2003;3(1–2):19–37. Epub 2003/08/12. . 12908228 10.1166/jnn.2003.189

[33] Dillon AC, Yudasaka M, Dresselhaus MS. Employing Raman spectroscopy to qualitatively evaluate the purity of carbon single-wall nanotube materials. J Nanosci Nanotechnol. 2004;4(7):691–703. Epub 2004/12/02. . 15570946 10.1166/jnn.2004.116

[34] Liu Z, Davis C, Cai W, He L, Chen X, Dai H. Circulation and long-term fate of functionalized, biocompatible single-walled carbon nanotubes in mice probed by Raman spectroscopy. Proc Natl Acad Sci U S A. 2008;105(5):1410–5. Epub 2008/01/31. 10.1073/pnas.0707654105 18230737 PMC2234157

[35] Al Faraj A, Fauvelle F, Luciani N, Lacroix G, Levy M, Cremillieux Y, et al *In vivo* biodistribution and biological impact of injected carbon nanotubes using magnetic resonance techniques. Int J Nanomedicine. 2011;6:351–61. Epub 2011/04/19. 10.2147/ijn.s16653 21499425 PMC3075901

[36] Kuzmany H, Plank W, Hulman M, Kramberger C, Grüneis A, Pichler T, et al Determination of SWCNT diameters from the Raman response of the radial breathing mode. Eur Phys J B. 2001;22(3):307–20.

[37] Shvedova AA, Kapralov AA, Feng WH, Kisin ER, Murray AR, Mercer RR, et al Impaired clearance and enhanced pulmonary inflammatory/fibrotic response to carbon nanotubes in myeloperoxidase-deficient mice. PLoS One. 2012;7(3):e30923 Epub 2012/04/06. 10.1371/journal.pone.0030923 22479306 PMC3316527

[38] Jones DP. Redefining oxidative stress. Antioxid Redox Signal. 2006;8(9–10):1865–79. Epub 2006/09/22. 10.1089/ars.2006.8.1865 . 16987039

[39] Crouzier D, Follot S, Gentilhomme E, Flahaut E, Arnaud R, Dabouis V, et al Carbon nanotubes induce inflammation but decrease the production of reactive oxygen species in lung. Toxicology. 2010;272(1–3):39–45. Epub 2010/04/13. 10.1016/j.tox.2010.04.001 . 20381574

[40] Fenoglio I, Tomatis M, Lison D, Muller J, Fonseca A, Nagy JB, et al Reactivity of carbon nanotubes: free radical generation or scavenging activity? Free Radic Biol Med. 2006;40(7):1227–33. Epub 2006/03/21. 10.1016/j.freeradbiomed.2005.11.010 . 16545691

[41] Ren L, Zhong W. Oxidation reactions mediated by single-walled carbon nanotubes in aqueous solution. Environ Sci Technol. 2010;44(18):6954–8. Epub 2010/08/19. 10.1021/es101821m . 20715868

[42] Kotchey GP, Gaugler JA, Kapralov AA, Kagan VE, Star A. Effect of antioxidants on enzyme-catalysed biodegradation of carbon nanotubes. J Mater Chem B Mater Biol Med. 2013;1(3):302–9. Epub 2013/04/30. 10.1039/c2tb00047d 23626907 PMC3634595

[43] Maher P. Redox control of neural function: background, mechanisms, and significance. Antioxid Redox Signal. 2006;8(11–12):1941–70. Epub 2006/10/13. 10.1089/ars.2006.8.1941 . 17034341

[44] Kobayashi M, Yamamoto M. Molecular mechanisms activating the Nrf2-Keap1 pathway of antioxidant gene regulation. Antioxid Redox Signal. 2005;7(3–4):385–94. Epub 2005/02/12. 10.1089/ars.2005.7.385 . 15706085

[45] Cai X, Jia H, Liu Z, Hou B, Luo C, Feng Z, et al Polyhydroxylated fullerene derivative C(60)(OH)(24) prevents mitochondrial dysfunction and oxidative damage in an MPP(+)-induced cellular model of Parkinson's disease. J Neurosci Res. 2008;86(16):3622–34. Epub 2008/08/19. 10.1002/jnr.21805 . 18709653

[46] da Rocha AM, Ferreira JR, Barros DM, Pereira TC, Bogo MR, Oliveira S, et al Gene expression and biochemical responses in brain of zebrafish *Danio rerio* exposed to organic nanomaterials: carbon nanotubes (SWCNT) and fullerenol (C60(OH)18-22(OK4)). Comp Biochem Physiol A Mol Integr Physiol. 2013;165(4):460–7. Epub 2013/04/02. 10.1016/j.cbpa.2013.03.025 . 23542748

